# Potential of selected lactic acid bacteria from *Theobroma cacao* fermented fruit juice and cell-free supernatants from cultures as inhibitors of *Helicobacter pylori* and as good probiotic

**DOI:** 10.1186/s13104-020-4923-7

**Published:** 2020-02-10

**Authors:** Laure Brigitte Kouitcheu Mabeku, Samuel Ngue, Idris Bonsou Nguemo, Hubert Leundji

**Affiliations:** 1 Microbiology and Pharmacology Laboratory, Department of Biochemistry, Faculty of Science, University of Dschang, P. O. Box 67 Dschang, Cameroon; 2 Gastroenterology Department, Laquintinie Hospital of Douala, P. O. Box 4035 Douala, Cameroon

**Keywords:** Lactic acid bacteria, *Theobroma cacao*, Antimicrobial activity, *Helicobacter pylori*, Probiotic

## Abstract

**Objective:**

The present study was designed to evaluate the inhibitory effect of lactic acid bacteria (LAB) isolates from the fermented cocoa juice and their cell-free culture supernatants (CFS) against *Helicobacter pylori* strains and their potential as good probiotic. Isolation of lactic acid bacteria (LAB) was performed by culture and subculture of sample on MRS agar. Morphological characteristics, Gram staining and catalase reaction were used to identify the isolates. The antagonistic activity of LAB was tested using the agar spot-on-lawn method and the inhibitory effect of CFS using well diffusion assay. Acid tolerance and resistance to antibiotics tests were used to evaluate the probiotic potential of LAB isolates.

**Results:**

Antagonistic effect was observed in 65.52% of isolated LAB. Isolate LAB19 showed the broader spectrum of antagonistic effect. The overall inhibitory activity was two to three folds reduced when CFSs were used instead of LAB isolates themselves. Our data showed that LAB19 controlled *H. pylori* growth using bacteriocins and that LAB4′, LAB8, LAB11′, LAB12, LAB13′, LAB15, LAB16 and LAB17 were through organic acids. LAB9, LAB11′ and LAB12 showed properties of probiotic tested. In this study, nine LAB isolates were found to possess anti-*Helicobacter* activity and some preliminary probiotic properties.

## Introduction

*Helicobacter pylori* is one of the most common agent implicated in bacterial infection worldwide [1]. The presence of *H. pylori* in the gastric lining causes gastritis which might lead to more disastrous ulceration or malignant tumors [1]. Treatment options for curing *H. pylori* infection included, triple therapy which consist of an acid suppressor with clarithromycin, amoxicillin, or nitroimidazolic compounds, taken over a period of 7–14 days [2]. In case the triple therapy failure, the quadruple therapy is administered [3]. Despite this fact, it has been noticed that the eradication rate of this micro-organism varies from 65 to 80%. Factors such as; disrespect of the medical prescription, bacterial resistance and adverse antibiotics effects are responsible for this treatment failure [4, 5]. In a bid to improve the treatment tolerability and eradication rate of this infection, several strategies are needed [6], among which the use of the probiotics [2]. Most probiotics inhibit the growth of pathogens and produce beneficial fermentation products such as short chain fatty acids **[**7, 8]. Many probiotic species produce vitamins and useful enzymes and help to maintain gut health. A few probiotic strains have immune, neurological effects. Various probiotic mixtures have been evaluated for their efficacy in improving the treatment of *H. pylori* infection and preventing side effects from treatment [9–13] and the results are variable [14]. In this work, we focused on the inhibitory effects of a collection of lactic acid bacteria (LAB) isolated from fermented cocoa juice and their metabolites against *H. pylori* strains. The potential of active LAB isolates as good probiotic were also evaluated.

## Main text

### Methods

#### Bacterial strains tested for inhibition

Eight *H. pylori* clinical strains (Hp0011, Hp0012, Hp0013, Hp0014, Hp0015, Hp0016, Hp00116 and HP00117) were used in this study. The strains were isolated from patients with gastro duodenal disorders requiring gastroendoscopic examination at Laquintinie Hospital, Douala, Cameroon. All *H. pylori* strains were grown at 37 °C on supplemented Columbia agar (Columbia agar + 5% (v/v) lacked horse blood and 1% (v/v) Vitox) for 48 to 72 h under microaerophilic conditions.

#### Sample collection

The source of lactic acid bacteria used were fermented cocoa juice. Cocoa pods were collected from cocoa plantation in the Littoral region of Cameroon. The contain of cocoa pods were aseptically remove and kept in sterile plastic jar for 48 h at room temperature for fermentation.

#### Isolation and identification of lactic acid bacteria (LAB)

Ten g of the sample was inserted into a 90 ml sterile phosphate-buffered saline (PBS 0.1 M, 0.8% NaCl, pH- 7.2) and homogenized. After this, 0.1 ml of the sixth tenfold serial dilutions of homogenized samples were spread on MRS agar and incubated at two different temperatures, 30 °C and 37 °C for 48 h under anaerobic conditions [15]. The colonies gotten from this were then sub-cultured trice on a new MRS agar in order to have single pure colonies [16]. The purified single colonies were then selected at randomly and cultured in MRS broth at 37 °C for 24 h in aerobiosis. The next step was the morphological characterization (size, color, edge form and texture of the colony), Gram staining and catalase reaction for the identification of the isolates [17, 18]. Purified lactic acid bacteria (LAB) were those that were both positive to the Gram staining technique but negative to the catalase reaction. Pellets from the MRS broth LAB cultures were re-suspended in broth containing 15% glycerol, and aliquots were frozen for further use.

#### Antagonistic activity of LAB isolates

In order to determine the antagonistic activity we used the agar spot-on-lawn method where the isolated LAB was tested against *H. pylori* [19]. An aliquot (2 µl) of an overnight LAB culture was spotted onto MRS agar plates and incubated anaerobically at 37 °C for 48 h. Control plates were prepared with MRS agar only. The plates were subsequently overlaid with soft supplemented Columbia Agar containing 2% *H. pylori* strains. Plates were incubated aerobically at 37 °C for 24 h. The susceptibility of the same pathogenic strains to a range of antibiotics used as positive control was reported in our previous study [20]. The test was done in triplicate. After incubation, the inhibition zone around the LAB spot was noticed and the results were expressed as a mean inhibition zone and standard error.

### Test of cell-free supernatants (CFS) inhibitory effect

#### Preparation of CFSs

Only LAB isolates showing antagonistic effect against *Helicobacter pylori* strains tested were selected. They were grown individually in 20 ml MRS broth and incubating anaerobically at 37 °C for 15 h. The supernatant was recovered by centrifugation at 3500 rpm for 1 h. The cell free supernatants (CFSs) were obtained by passing the supernatants through 0.4 μm pore size filters.

#### Test of CFS inhibitory effect: well diffusion assay

*Helicobacter. pylori* cultures were plated on fresh supplemented Colombia agar plates (10^7^ CFU per plate), and wells were drilled into the agar. 50 µl aliquots of each native cell-free culture supernatants were suspended in the agar wells. 50 µl of MRS broth (pH 6.0) were also suspended in the agar wells and taken as control wells. The test was done in triplicate. Plates were incubated for 48 to 72 h under microaerophilic conditions at 37 °C, the diameters of inhibition zones around the wells were measured and expressed as mean diameter and standard error.

### Characterization of active components of CFCs

#### Cell-free culture supernatant treatment

Native active CFSs were subjected to three treatments before evaluating their inhibitory activity against *H. pylori* using well diffusion assay as described above. Heat treatment and pH adjustment to determine respectively if protein or acid component was required for their bactericidal activity and to both pH adjustment and heat treatment for confirmation. For this purpose, an individual portion of each supernatant was adjusted to pH 6.5–6.8 with NaOH, heat-treated (100 °C for 1 h) and both boiled for 1 h and adjusted to pH 6.5–6.8 with NaOH.

### Evaluation of probiotic potential of active LAB isolates

#### Acid tolerance

The previously described technique was used to find out the resistance of the isolated LAB to the acidic gastric pH [21]. Pure isolates were inoculated in MRS broth and incubated at 37 for 18 h. The 18 h bacterial cultures were centrifuged at 3500 rpm for 20 min and the pellets were washed in sterile phosphate-buffered saline (0.1 M phosphate buffer, 0.8% NaCl, pH- 7.2) and resuspended in 1 ml of PBS (pH- 7.2). From this, 0.1 ml was added to 10 ml of MRS whose pH had been adjusted to 2 or 7 using 1 N HCl or NaOH. Bacterial growth was monitored by determination of optical density at 620 nm after 0, 3, 6 and 24 h incubation period at 37 °C. The surviving percent was calculated as follows:

$$ {\text{Surviving}}\left( \% \right) = \frac{{\Delta {\text{DO pH  }}7 - \Delta {\text{DO pH  }}2}}{{\Delta {\text{DO pH  }}7}} \times { 1}00 $$

(∆DO pH7), (∆DO pH2): optical density at pH 7.0 and pH 2. An isolate with a surviving percent up to 50% was considered as surviving. They were then classified as excellent if the isolate survived at pH 2 after 24 h; very good after 6 h; good after 3 h and poor before 3 h.

#### Resistance to antibiotics

The agar overlay diffusion method [22] was used to find out the susceptibility of the selected LAB isolates against amoxicillin (30 µg), erythromycin (15 µg), chloramphenicol (30 µg) and imipenem (10 µg). The diameter of inhibition was noticed and the LAB isolates were further categorized as resistance, intermediate or susceptible [23, 24].

## Results

### Isolation and identification of lactic acid bacteria (LAB)

Fifty strains were purified from the above fermented food and 29 (58%) were characterized as LAB.

### Antagonistic activity of isolated LAB

Antagonistic effect was noticed in 65.52% (19/29) of the tested LAB isolates with inhibition zones ranging from 6 to 30 mm (Table 1). The highest inhibition zone (30 mm) was obtained with isolates LAB4′, LAB6, LAB11 and LAB19 against 12.5 to 25% of the tested pathogen. The spectrum of inhibitory activity ranged from 50 to 100% with that of isolate BL19 as the broader one. The most susceptible pathogenic strain to LAB isolates detected was HP0014 whereas HP00117 was the most resistant.

**Table 1:** Antagonistic activity of isolated LAB (19) against *H. pylori* clinical strains (08) tested (mm)

Lactic acid bacteria isolates	*H. pylori* strains								Antagonistic effect (%)
	Hp 0011	Hp 0012	Hp 0013	Hp 0014	Hp 00115	Hp 0016	Hp 00116	Hp 00117	
LAB1	–	–	22	24	12	–	14	–	50
LAB2	–	–	22	17	8	–	10	–	50
LAB3	–	–	25	18	9	–	7	–	50
LAB4	–	–	24	23	12	–	8	–	50
LAB4′	28	–	30	16	–	30	7	–	62.5
LAB6	26	–	16	20	–	30	–	–	50
LAB8	27	–	20	12	–	20	9	–	62.5
LAB9	22	–	17	6	–	20	11	–	62.5
LAB11	–	15	30	14	14	–	–	–	50
LAB11′	16	20	15	12	10	–	12	–	75
LAB12	22	26	15	16	12	–	14	–	75
LAB13	11	–	–	14	–	14	–	–	50
LAB13′	12	15	–	17	15	14	–	–	62.5
LAB15	27	15	18	16	25	–	22	16	87.5
LAB16	25	20	21	17	–	–	–	–	50
LAB17	24	20	25	27	22	16	–	8	87.5
LAB19	30	24	14	18	25	22	23	18	100
LAB23	16	9	–	16	14	–	–	–	50
LAB31	19	17	21	–	18	–	–	–	50
Susceptibility (%)	73.68	52.63	84.21	94.73	68.42	42.10	63.15	15.78	

Each value represents the mean of three determination

(–) no activity

HP, *Helicobacter pylori;* LAB, lactic acid bacteria

### Test for cell-free supernatants (CFS) inhibitory effect

**Fig. 1:**
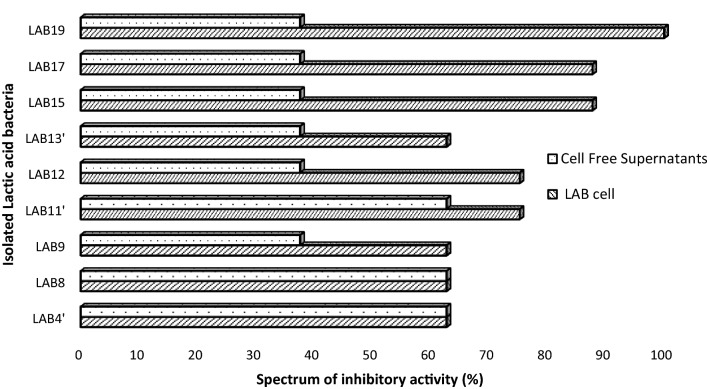
Spectrum of inhibitory activity of selected LAB and their corresponding cell free supernatant

The inhibitory activity of CFS from 47% (9/19) of the antagonistic LAB isolates were evaluated (Table 2). Our data show that the presence of these cell free culture supernatants reduced the viability of the pathogens strains. The overall inhibitory activity was two- to three folds reduced when CFSs were used instead of LAB isolates themselves (Fig. 1). The inhibition zone ranging from 5 to 10 mm with CFSs instead of 6 to 30 mm with LAB isolates. Similarly, the spectrum of inhibitory activity varying from 37.5 to 62.5% with CFSs instead of 62.5 to 100% with LAB isolates. Derived from active LAB; CFSs-LAB4′, -LAB8, -LAB9, -LAB12, -LAB13′, -LAB15, -LAB17 and -LAB19 did not show any inhibitory activity.

**Table 2:** Diameter of inhibitory effect of cell free culture supernatants (CFSs) from LAB isolates with broader spectrum of inhibitory activity (9) against *H. pylori* clinical strains (08) tested (mm)

*Helicobacter. pylori* strains	Native cell free culture supernatants from selected LAB isolates									Susceptibility (%)
	CFS-LAB4′	CFS-LAB8	CFS-BL9	CFS-LAB11′	CFS-LAB12	CFS-LAB13′	CFS-LAB15	CFS-LAB17	CFS-LAB19	
Hp 0011	10	8	–	6	–	–	–	–	–	33.33
Hp 0012	7	8	–	6	–	–	–	–	–	33.33
Hp 0013	8	10	7	5	4	4	6	5	8	100
Hp 0014	8	8	6	7	5	3	7	6	8.5	100
Hp00115	7	8	5	6	6	4	6	7	8	100
Hp 0016	–	–	–	–	–	–	–	–	–	0
Hp 00116	–	–	–	–	–	–	–	–	–	0
Hp 00117	–	–	–	–	–	–	–	–	–	0
Inhibitory activity (%)	62.5	62.5	37.5	62.5	37.5	37.5	37.5	37.5	37.5	

Each value represents the mean of three determination

(–) no activity

CFS, cell free culture supernatants; HP, *Helicobacter pylori;* LAB, lactic acid bacteria

### Characterization of active component of cell-free supernatants (CFSs)

The pH of the native supernatants before pH adjustment were in the range of 4.5–4.8, with 3.8 as the average pH value, which was lower than fresh MRS broth (pH 6.0). Instead of the others CFSs, no significant difference was found between activity of the native and neutralized CFS from LAB19 cells (Additional file 1). Moreover, only CFS from BL19 lost its activity with heat treatment (Additional file 2). All the selected CFSs lost their bactericidal activity with pH adjustment and heat treatment (Additional file 3).

### Probiotic potential of active LAB isolates

All the tested LAB isolates showed at least 50% cells survival at pH 2 after incubation up to 3 h. LAB9′ and LAB11 isolates demonstrated a tolerance to acidic pH after incubation for 6 h and up to 24 h for LAB12 (Additional file 4). They were also susceptible to antibiotics used with diameter of inhibition zones ranging from 23 to 44 mm (Additional file 5).

## Discussion

In this work, some isolated LAB showed an antagonistic effect against the tested pathogenic strains. Inhibitory effect was much stronger in LAB and *H. pylori* co-cultures rather than cell cultured free supernatants and *H. pylori*. Moreover, some CFSs derived from an active LAB isolate did not show any inhibitory activity, suggesting that the isolated bacteria itself displays killing activity. Enany et al. has demonstrated that in vitro, *Lactobacillus acidophilus* inhibits *H. pylori* growth [25]. In *H. pylori* colonized Mongolian gerbils, gastric perfusion with *Lactobacillus* strains clear *H pylori* colonization in their stomach with the clearance rate of about 60% [26]. Also, previous studies reported that the use of probiotics combined to standard therapy in *H. pylori* infected patients, increased the eradication rate of the organism and decreased the overall rate of adverse events [12, 27]. This antagonistic effect might be carried out through reduction in urease activity mediated by short-chain fatty acids produced by probiotics, an enhancement of the acidic environment of the stomach, damages of the cell wall of *H. pylori* strains, and inhibition of the colonization of *H. pylori* in the gastric mucosa [28–30]. In contrast, in a meta-analysis, different mixtures of probiotics species and strains didn’t improve eradication rates of *H. pylori*, but decrease the side effects resulting from therapy [31]. However, when examining *Saccharomyces boulardii* CNCM I-745 only, eradication rates and adverse symptoms were improved [32].

Our finding also showed that some LAB isolates secreted active compounds in the culture medium. It is reported that in an adequate broth medium, some LAB secrete organic acids, hydrogen peroxide, and bacteriocins to antagonize pathogen growth [33–36]. Further characterization showed that CFS from LAB 19 inhibits *H. pylori* growth through the production of bacteriocin, and that the other isolates act through the production of compounds such as organic acids [37, 38].

Our finding also revealed that LAB 9, LAB 11′ and LAB 12 isolate are potential probiotic since they are capable of withstanding at the low gastric pH [39] and may not serve as host of antibiotic resistance genes [40–42].

## Limitations

Further phenotypic and genotypic characterization of the isolated LAB as probiotic and anti-*Helicobacter* activity are necessary in order to elucidate their potential beneficial health effects.

## Supplementary information

**Additional file 1.** Effect of pH adjustment on the inhibitory effect of cell free culture supernatants (CFSs) against *H. pylori* clinical strains (08) tested (mm).

**Additional file 2.** Effect of heat treatment on the inhibitory effect of cell free culture supernatants (CFSs) against *H. pylori* clinical strains (08) tested (mm).

**Additional file 3.** Effect of heat treatment and pH adjustment on the inhibitory effect of cell free culture supernatants (CFSs) against *H. pylori* clinical strains (08) tested (mm).

**Additional file 4.** Resistance to acidic pH (pH = 2) of isolated lactic acid bacteria at different incubation times (3h, 6h and 24h).

**Additional file 5.** Susceptibility of isolates to antibiotics.

## Data Availability

The datasets used and/or analyzed during the current study are available from the corresponding author on reasonable request.

## Abbreviations

LAB: Lactic acid bacteria
CFS: Cell-free cultured supernatant
HP: *Helicobacter pylori*
