# Supplementary material for: Potential of selected lactic acid bacteria from Theobroma cacao fermented fruit juice and cell-free supernatants from cultures as inhibitors of Helicobacter pylori and as good probiotic
Source: BMC Res Notes. 2020 Feb 10;13:64. doi: 10.1186/s13104-020-4923-7 (PMC7011242; doi:10.1186/s13104-020-4923-7)
Supplement: Supplementary file 1 — Additional file 1. Effect of pH adjustment on the inhibitory effect of cell free culture supernatants (CFSs) against H. pylori clinical strains (08) tested (mm). [file 13104_2020_4923_MOESM1_ESM.docx]

**Additional file 1**

Effect of pH adjustment on the inhibitory effect of cell free culture supernatants (CFSs) against *H. pylori* clinical strains (08) tested (mm)

| ***H. pylori* strains** | **Neutralized cell free culture supernatants from selected LAB isolates** | | | | | | | | | **Susceptibility (%)** |
| --- | --- | --- | --- | --- | --- | --- | --- | --- | --- | --- |
|  | **CFS-LAB4’** | **CFS-LAB8** | **CFS-BL9** | **CFS-LAB11’** | **CFS-LAB12** | **CFS-LAB13’** | **CFS-LAB15** | **CFS-LAB17** | **CFS-LAB19** |  |
| Hp 0011 | 8 | - | -- | -- | -- | -- | -- | -- | -- | **11.11** |
| Hp 0012 | - | - | -- | -- | -- | -- | -- | -- | -- | **0.00** |
| Hp 0013 | 6 | 8 | -- | -- | -- | -- | -- | -- | 8 | **33.33** |
| Hp 0014 | 6 | 6 | -- | -- | -- | -- | -- | -- | 8.5 | **33.33** |
| Hp00115 | - | 6 | -- | -- | -- | -- | -- | -- | 8 | **22.22** |
| Hp 0016 | -- | -- | -- | -- | -- | -- | -- | -- | -- | **0** |
| Hp 00116 | -- | -- | -- | -- | -- | -- | -- | -- | -- | **0** |
| Hp 00117 | -- | -- | -- | -- | -- | -- | -- | -- | -- | **0** |
| **Inhibitory activity (%)** | **37.5** | **37.55** | **0** | **0** | **0** | **0** | **0** | **0** | **37.5** |  |

(-): no activity, CFS: cell free culture supernatants, HP: *Helicobacter pylori,* LAB: Lactic acid bacteria. Each value represents the mean of three determination.
