# Supplementary material for: Potential of selected lactic acid bacteria from Theobroma cacao fermented fruit juice and cell-free supernatants from cultures as inhibitors of Helicobacter pylori and as good probiotic
Source: BMC Res Notes. 2020 Feb 10;13:64. doi: 10.1186/s13104-020-4923-7 (PMC7011242; doi:10.1186/s13104-020-4923-7)
Supplement: Supplementary file 2 — Additional file 2. Effect of heat treatment on the inhibitory effect of cell free culture supernatants (CFSs) against H. pylori clinical strains (08) tested (mm). [file 13104_2020_4923_MOESM2_ESM.docx]

**Additional file 2**

Effect of heat treatment on the inhibitory effect of cell free culture supernatants (CFSs) against *H. pylori* clinical strains (08) tested (mm)

| ***H. pylori* strains** | **Heat treated cell free culture supernatants from selected LAB isolates** | | | | | | | | | **Susceptibility (%)** |
| --- | --- | --- | --- | --- | --- | --- | --- | --- | --- | --- |
|  | **CFS-LAB4’** | **CFS-LAB8** | **CFS-BL9** | **CFS-LAB11’** | **CFS-LAB12** | **CFS-LAB13’** | **CFS-LAB15** | **CFS-LAB17** | **CFS-LAB19** |  |
| Hp 0011 | 7 | 7 | -- | 6 | -- | -- | -- | -- | -- | **33,33** |
| Hp 0012 | 8 | 7 | -- | 6 | -- | -- | -- | -- | -- | **33,33** |
| Hp 0013 | 9 | 10 | 8 | 5 | 6 | 6 | 8.5 | 9 | -- | **88,88** |
| Hp 0014 | 10 | 8 | 6 | 7 | 4 | 4 | 7 | 8 | -- | **88,88** |
| Hp00115 | 8 | 9 | 6 | 6 | 6 | 5 | 7.5 | 8 | -- | **88,88** |
| Hp 0016 | -- | -- | -- | -- | -- | -- | -- | -- | -- | **0** |
| Hp 00116 | -- | -- | -- | -- | -- | -- | -- | -- | -- | **0** |
| Hp 00117 | -- | -- | -- | -- | -- | -- | -- | -- | -- | **0** |
| **Inhibitory activity (%)** | **62.5** | **62.5** | **37.5** | **62.5** | **37.5** | **37.5** | **37.5** | **37.5** | **0** |  |

(-): no activity, CFS: cell free culture supernatants, HP: *Helicobacter pylori,* LAB: Lactic acid bacteria. Each value represents the mean of three determination.
