# Supplementary material for: Potential of selected lactic acid bacteria from Theobroma cacao fermented fruit juice and cell-free supernatants from cultures as inhibitors of Helicobacter pylori and as good probiotic
Source: BMC Res Notes. 2020 Feb 10;13:64. doi: 10.1186/s13104-020-4923-7 (PMC7011242; doi:10.1186/s13104-020-4923-7)
Supplement: Supplementary file 3 — Additional file 3. Effect of heat treatment and pH adjustment on the inhibitory effect of cell free culture supernatants (CFSs) against H. pylori clinical strains (08) tested (mm). [file 13104_2020_4923_MOESM3_ESM.docx]

**Additional file 3**

Effect of heat treatment and pH adjustment on the inhibitory effect of cell free culture supernatants (CFSs) against *H. pylori* clinical strains (08) tested (mm)

| ***H. pylori* strains** | **Cell free culture supernatants from selected LAB isolates** | | | | | | | | | **Susceptibility (%)** |
| --- | --- | --- | --- | --- | --- | --- | --- | --- | --- | --- |
|  | **CFS-LAB4’** | **CFS-LAB8** | **CFS-BL9** | **CFS-LAB11’** | **CFS-LAB12** | **CFS-LAB13’** | **CFS-LAB15** | **CFS-LAB17** | **CFS-LAB19** |  |
| Hp 0011 | -- | -- | -- | -- | -- | -- | -- | -- | -- | **0** |
| Hp 0012 | -- | -- | -- | -- | -- | -- | -- | -- | -- | **0** |
| Hp 0013 | -- | -- | -- | -- | -- | -- | -- | -- | -- | **0** |
| Hp 0014 | -- | -- | -- | -- | -- | -- | -- | -- | -- | **0** |
| Hp00115 | -- | -- | -- | -- | -- | -- | -- | -- | -- | **0** |
| Hp 0016 | -- | -- | -- | -- | -- | -- | -- | -- | -- | **0** |
| Hp 00116 | -- | -- | -- | -- | -- | -- | -- | -- | -- | **0** |
| Hp 00117 | -- | -- | -- | -- | -- | -- | -- | -- | -- | **0** |
| **Inhibitory activity (%)** | **0** | **0** | **0** | **0** | **0** | **0** | **0** | **0** | -- |  |

(--): no activity, CFS: cell free culture supernatants, HP: *Helicobacter pylori,* LAB: Lactic acid bacteria. Each value represents the mean of three determination.
