# Supplementary material for: Potential of selected lactic acid bacteria from Theobroma cacao fermented fruit juice and cell-free supernatants from cultures as inhibitors of Helicobacter pylori and as good probiotic
Source: BMC Res Notes. 2020 Feb 10;13:64. doi: 10.1186/s13104-020-4923-7 (PMC7011242; doi:10.1186/s13104-020-4923-7)
Supplement: Supplementary file 4 — Additional file 4. Resistance to acidic pH (pH = 2) of isolated lactic acid bacteria at different incubation times (3h, 6h and 24h). [file 13104_2020_4923_MOESM4_ESM.docx]

**Additional file 4**

Figure 2: Resistance to acidic pH (pH = 2) of isolated lactic acid bacteria at different incubation times (3h, 6h and 24h).
