# Supplementary material for: Potential of selected lactic acid bacteria from Theobroma cacao fermented fruit juice and cell-free supernatants from cultures as inhibitors of Helicobacter pylori and as good probiotic
Source: BMC Res Notes. 2020 Feb 10;13:64. doi: 10.1186/s13104-020-4923-7 (PMC7011242; doi:10.1186/s13104-020-4923-7)
Supplement: Supplementary file 5 — Additional file 5. Susceptibility of isolates to antibiotics. [file 13104_2020_4923_MOESM5_ESM.docx]

**Additional file 5**

Susceptibility of isolates to antibiotics

| **Lactic acid bacteria (LAB) isolates** | Antibiotics | | | | Resistance (%) |
| --- | --- | --- | --- | --- | --- |
|  | Amoxicillin (30 µg) | Erythromycin (15 µg) | Chloramphénicol (30 µg) | Imipenem (10 µg) |  |
| LAB4’ | 30.50 ± 0.70 | 25.50 ± 3.53 | 35.00 ± 4.24 | 34.00 ± 5.65 | 0.00 |
| LAB 8 | 38.00 ± 2.82 | 26.50 ± 4.94 | 35.00 ± 0.00 | 41.00 ± 1.41 | 0.00 |
| LAB 9 | 26.50 ± 6.36 | 24.50 ± 6.36 | 26.50 ± 4.94 | 29.00 ± 5.65 | 0.00 |
| LAB 11’ | 30.00 ± 0.00 | 26.50 ± 0.70 | 32.00 ± 0.00 | 40.00 ± 0.00 | 0.00 |
| LAB 12 | 40.00 ± 0.00 | 29.50 ± 0.70 | 32.50 ± 3.53 | 40.00 ± 0.00 | 0.00 |
| LAB 13’ | 41.00 ± 0.00 | 24.00 ± 2.82 | 30.50 ± 0.70 | 39.00 ± 0.00 | 0.00 |
| LAB 15 | 40.00 ± 0.00 | 26.00 ± 1.41 | 31.00 ± 1.41 | 44.00 ± 1.41 | 0.00 |
| LAB 17 | 40.00 ± 0.00 | 29.50 ± 0.70 | 32.50 ± 0.70 | 42.00 ± 0.00 | 0.00 |
| LAB 19 | 38.50 ± 2.12 | 23.50 ± 2.12 | 30.00 ± 0.00 | 40.00 ± 0.00 | 0.00 |

LAB: Lactic acid bacteria. Each value represents the mean of three determination.
